# Isolate-level pathogenic heterogeneity in keratitis caused by the *Fusarium solani* species complex: corneal invasion, host immunopathology, and model fidelity

**DOI:** 10.3389/fcimb.2026.1984688

**Published:** 2026-10-22

**Authors:** Xin He, Ze Li, Shifei Wei, Yunyun Sun, Wenzai An, Qinyi Cui, Tao Wang, Qiuli Ming, Ningli Wang

**Affiliations:** 1 The First Affiliated Hospital, and College of Clinical Medicine of Henan University of Science and Technology, Luoyang, Henan, China; 2 Henan Academy of Innovations in Medical Science Eye Institute, Zhengzhou, Henan, China; 3 Department of Clinical Laboratory, The Fifth Affiliated Hospital of Zhengzhou University, Zhengzhou, Henan, China; 4 Beijing Tongren Eye Center, Beijing Tongren Hospital, Capital Medical University, Beijing, China; 5 Beijing Aier-Intech Eye Hospital, Beijing, China; 6 The Third Affiliated Hospital of Henan Medical University, Xinxiang, Henan, China; 7 National Engineering Research Center for Ophthalmic Equipment, Beijing, China; 8 Beijing Institute of Ophthalmology, Beijing Tongren Eye Center, Beijing Tongren Hospital, Capital Medical University, Beijing, China

**Keywords:** corneal invasion, experimental models, fungal keratitis, *Fusarium solani* species complex, host–pathogen interaction, immunopathology, isolate heterogeneity

## Abstract

Fungal keratitis caused by the *Fusarium solani* species complex (FSSC) often progresses despite antifungal therapy and can result in irreversible visual loss. Yet biological diversity within FSSC has not been integrated across taxonomy, isolate phenotype, corneal models, host response, and clinical outcome. This Review synthesizes taxonomic, experimental, and clinical evidence on isolate-level variation in adhesion, epithelial entry, stromal invasion, biofilm formation, tissue damage, immune activation, and antifungal response. Available data support substantial isolate-dependent heterogeneity but do not justify a stable species- or member-level ranking of clinical severity. Fungal developmental state, inoculation route, epithelial integrity, stromal architecture, host immunity, treatment timing, and tissue drug exposure all modify the phenotype observed. Cell, tissue, animal, and human-specimen models therefore provide complementary rather than interchangeable evidence, and few studies carry the same authenticated clinical isolate from corneal testing to longitudinal patient outcomes. Refractory FSSC keratitis is best investigated through a matched isolate–model–patient approach integrating multilocus authentication, spatial fungal localization, viable burden, host-mediated injury, compartment-specific drug exposure, and prespecified clinical endpoints. Such designs could distinguish persistent infection from inadequate tissue exposure and immunopathology and thereby improve risk stratification and therapeutic development.

## 1 Introduction

Fungal keratitis accounts for a disproportionate share of avoidable monocular blindness, particularly in tropical, resource-constrained, and agricultural settings, where *Fusarium* is a leading filamentous fungal pathogen (Brown et al., 2022). Even within specialist European services, delayed recognition, prolonged antifungal courses, limited visual recovery, and resort to surgery remain common (Lamas-Francis et al., 2024). A two-decade Canadian multicenter series describes the same pattern in a high-income temperate setting: severe disease, protracted treatment, corneal surgery, and permanent visual loss (Ling et al., 2024). Microbiological confirmation often arrives too late to guide early management (Toumasis et al., 2024). Randomized trials have tested only a small set of antifungal regimens (Farnan et al., 2026), while drug delivery to the cornea poses a separate, compartment-specific pharmacological challenge. Within the avascular stroma, fungal proliferation, extracellular-matrix degradation, and host-mediated injury converge to produce corneal melt, deep extension, and perforation; culture burden alone does not capture this process (Abbondante et al., 2023; García-López et al., 2023).

The historical label *Fusarium solani* encompasses deeply divergent lineages, and whether some of these fungi belong in *Fusarium* or *Neocosmospora* remains contested (Crous et al., 2021). Morphology and single-locus sequencing often stop short of reliable species-level resolution; multilocus sequencing against curated reference databases provides a firmer basis for identifying clinical isolates (Sáenz et al., 2024). Molecular analysis of keratitis specimens has exposed uncommon fusarioid taxa hidden beneath routine labels, illustrating in individual cases how a broad genus-level assignment can obscure the infecting organism (Monteiro et al., 2025). *In vitro* susceptibility also varies among corneal isolates, although available laboratory data and small clinical series do not support a taxonomic ordering of treatment response (Kang et al., 2025). Multi-omics studies extend this heterogeneity to accessory genomes, secondary metabolism, and environmental adaptation among closely related *Fusarium* strains, including strains assigned to the same species (Bugingo et al., 2025). Comparisons across the keratitis literature remain constrained by inconsistent identification methods, competing taxonomic schemes, and nonuniform outcome definitions (Cintra et al., 2024).

Recent three-dimensional and ex vivo corneal systems retain sufficient tissue architecture to examine adhesion, penetration, matrix disruption, and local host responses beyond the limits of monolayer culture (Cheng et al., 2024). Human corneal transcriptomics distinguished herpes simplex virus keratitis from a combined bacterial/fungal response group but offered little resolution specific to *Fusarium* (Lapp et al., 2024). Therapeutic keratoplasty specimens positive for *Fusarium* or *Aspergillus flavus* revealed shared and pathogen-associated inflammatory programs in a small cohort with advanced, previously treated disease (Dinesh et al., 2025). In an *F. solani* cell-and-mouse platform, perturbing Z-DNA-binding protein 1 (ZBP1) connected macrophage PANoptosis with immune escape, extracellular nucleic acids, and biofilm-associated biology (Gao et al., 2026). A separate murine study found that depletion of 6-phosphofructo-2-kinase/fructose-2,6-bisphosphatase 3 (PFKFB3) intensified phosphoinositide 3-kinase/protein kinase B (PI3K/AKT)- and nuclear factor kappa B (NF-κB)-associated inflammation, worsened corneal injury, and failed to improve fungal clearance (Zhang et al., 2025b). *Aspergillus fumigatus* comparator studies linked epithelial exosomal signaling to macrophage activation and reported an anti-inflammatory effect of isoimperatorin within that experimental setting (Yu and Wu, 2025; Song et al., 2026). The contribution of each design is distinct. Structured corneal models resolve spatial behavior, late surgical tissue captures host responses after treatment, and perturbation studies test pathway dependence. Authenticated isolate identity, early corneal behavior, tissue drug exposure, and patient outcome remain disconnected across these literatures.

Most fungal-keratitis reviews organize evidence around etiopathology, epidemiology, diagnosis, treatment, drug delivery, and adjunctive care (Bisen et al., 2024; Ghenciu et al., 2024). Diagnostic reviews summarize conventional and molecular methods, tear β-D-glucan has been evaluated as a rapid host-fluid biomarker, and imaging syntheses describe lesion morphology and depth (Cabrera-Aguas and Watson, 2023; Tawde et al., 2024). These approaches establish organism identity or disease location; isolate-specific pathogenic behavior requires functional testing. Taxonomy, fungal phenotype, host response, treatment, and model design have largely developed as separate literatures. This Review first establishes which isolate was studied, then follows its behavior across corneal systems and asks which features of human disease each system retains. Viewed in this way, keratitis caused by the *Fusarium solani* species complex (FSSC) is an isolate-dependent fungal–corneal interaction in which invasive phenotype, host immunopathology, and model fidelity together influence disease severity and treatment failure.

## 2 Taxonomic resolution and isolate diversity in FSSC keratitis

### 2.1 Nomenclature and species-complex assignment

Shifts in fungal nomenclature affect laboratory reporting, clinical communication, and the comparability of FSSC keratitis studies published decades apart (de Hoog et al., 2023). During periods of transition, reports should pair the updated name with the designation familiar to clinicians (Borman et al., 2025). Curated nomenclature databases and synonym records connect the label used in an original report with its current taxonomic assignment (Wang et al., 2023b). Multilocus analysis of the contact lens-associated outbreak uncovered substantial phylogenetic diversity among isolates reported broadly as *Fusarium* or historically as *Fusarium solani* (O’Donnell et al., 2007). For each study, the original organism name and identification method are retained, with a current designation added when supported by sequence data. Genus-level reassignment leaves the underlying biological observation unchanged even though taxonomic systems delimit *Fusarium* and *Neocosmospora* differently (Crous et al., 2021). Consistent terminology should also distinguish the pathogen, the disease, and the clinical syndrome, which are separate scientific entities (de Hoog et al., 2024).

Contemporary clinical-mycology classifications separate *Fusarium* from *Neocosmospora*, while historical FSSC reports may include lineages assigned differently under competing taxonomic systems (Sáenz et al., 2024). The one fungus, one name principle reduces dual nomenclature but cannot recover the identification resolution missing from older studies (Unalan-Altintop and Arikan-Akdagli, 2025). Each name change should be documented with the original label, current designation, identification method, and achieved resolution (Kidd et al., 2023). Without sequence data, a historical designation is best retained at the level supported by morphology or a single locus. This preserves continuity when the same strain appears under different names in clinical, taxonomic, and experimental publications. A species-complex designation identifies the group being compared. Adhesion, epithelial entry, stromal invasion, tissue damage, drug response, and clinical severity still require direct functional evidence. Taxonomy sets the population for study; pathogenicity is resolved at isolate level.

### 2.2 Authentication of clinical isolates

Isolate authentication should be performed at the resolution required by the proposed taxonomic or functional comparison. Sequence-based identification of *Fusarium* rests on informative loci, sequence quality, curated reference data, and an explicit assignment rule; FUSARIUM-ID v.3.0 adds versioned TEF1 resources and isolate metadata (O’Donnell et al., 2022; Torres-Cruz et al., 2022; Sáenz et al., 2024). Using sequential sequencing of the internal transcribed spacer (ITS), translation elongation factor 1-alpha (TEF1-α), and RNA polymerase II second largest subunit (RPB2), a multicenter Taiwanese series resolved more than 90% of clinical isolates to species level before susceptibility testing (Chen et al., 2026a). Clinical and hospital-environmental isolates require separate provenance categories even when ITS and TEF1-α sequencing places them in related phylogenetic clusters; phylogenetic proximity alone does not establish a common source (Chen et al., 2025). **Table 1** summarizes the authentication strategies and inferential limits of seven ocular studies. In a prospective Brazilian series, a concatenated TEF1-α and RPB2 phylogeny refined preliminary morphological and sequence matches, exposing the provisional nature of the initial labels (Ferreira da Cunha Neto et al., 2024). Interpretation depends on the reported locus, sequence quality, reference database and version, and phylogenetic or algorithmic method. A one-locus designation offers less resolution than a multilocus assignment or a validated algorithmic result.

**Table 1:** Taxonomic assignment and authentication of ocular fusarioid isolates.

Ocular isolate profile	Reported taxonomy	Authentication	Taxonomic resolution	Current interpretation	Main study contribution	Limitation	Reference
66 corneal isolates within 103 isolates	FSSC taxa reported as *Neocosmospora* spp.	ITS, TEF1-α, RPB2	>90% species level; one FSSC unresolved	FSSC retained; original names preserved	Stepwise multicenter identification workflow	Mixed specimens; no outcome linkage	(Chen et al., 2026a)
50 corneal isolates within 60 superficial isolates	*Neocosmospora* spp., FFSC, FOSC	Morphology, TEF1-α, RPB2	All isolates assigned to species	Original *Neocosmospora names* preserved	Phylogeny corrected preliminary assignments	Ten nonocular isolates; incomplete follow-up	(Ferreira da Cunha Neto et al., 2024)
27 isolates from 42 keratitis cases	FSSC, FFSC, FDSC	Morphology, TEF1-α, RPB2	14 FSSC isolates; 10 haplotypes	Reported names retained	Regional species–haplotype diversity	Selected subset; incomplete resolution	(Milanez et al., 2024)
29 corneal isolates	FSSC, FDSC, FFSC	Morphology, TEF1-α, MALDI-TOF MS	28 of 29 assigned to species	Reported *Fusarium* names retained	Expanded MALDI-TOF MS reference database	Single center; TEF1-α only	(Bagga et al., 2023)
52 isolates from 43 keratitis cases	Five complexes; 14 species	Morphology, ITS, TEF1-α	14 species; some complex-level assignments	Reported names retained	Linked identity, susceptibility, clinical features	Single center; heterogeneous treatment	(Huang et al., 2022b)
45 corneal isolates within 95 isolates	Seven complexes; named FSSC taxa	TEF1-α, MALDI-TOF MS	91.6% complex level; 11.6% species level	Reported names retained	Quantified the MALDI-TOF MS resolution gap	Mixed sources; limited libraries	(Guo et al., 2022)
91 corneal isolates within 319 isolates	FSSC, FOSC, FIESC haplotypes	RPB2, TEF1-α, nrRNA MLST	29 corneal haplotypes; 16 species	Historical haplotypes retained	Established outbreak phylogeny	Historical nomenclature; finite probes	(O’Donnell et al., 2007)

Taxonomic names are reported as used in the original studies. The “Current interpretation” column provides nomenclatural context and does not reassign isolates beyond the resolution supported by the original methods. Species-complex abbreviations are not italicized; genus and species names are italicized. FDSC, *Fusarium dimerum* species complex; FFSC, *Fusarium fujikuroi* species complex; FIESC, *Fusarium incarnatum-equiseti* species complex; FOSC, *Fusarium oxysporum* species complex; FSSC, *Fusarium solani* species complex; ITS, internal transcribed spacer; MALDI-TOF MS, matrix-assisted laser desorption/ionization time-of-flight mass spectrometry; MLST, multilocus sequence typing; nrRNA, nuclear ribosomal RNA; RPB2, RNA polymerase II second largest subunit; TEF1-α, translation elongation factor 1-alpha.

The performance of matrix-assisted laser desorption/ionization time-of-flight mass spectrometry (MALDI-TOF MS) depends heavily on the breadth and quality of its reference library. Among Southern Chinese clinical isolates, MALDI-TOF MS identified 91.6% to species-complex level and 11.6% to species level (Guo et al., 2022), while an expanded reference database in a South Indian keratitis series enabled correct species-level identification of 28 of 29 isolates (Bagga et al., 2023). In a separate mold evaluation, a molecularly assigned *F. solani* isolate was misidentified as *Rhizopus arrhizus*, supporting molecular confirmation when mass-spectrometric and sequence-based results conflict (Magrì et al., 2026). Multilocus sequencing combined with MALDI-TOF MS recovered uncommon fusarioid species from keratitis cases obscured by routine laboratory labels (Monteiro et al., 2025). A Taiwanese keratitis cohort linked molecular assignments with susceptibility and clinical variables but found no evidence that species-complex identity independently determined outcome (Huang et al., 2022b). A Mexican clinical-isolate study completed TEF1-α identification before susceptibility testing, retaining molecular identity alongside isolate-specific minimum inhibitory concentration (MIC) data (Román-Montes et al., 2025). The isolate record should include specimen source, a unique identifier or accession, original designation, identification method, database and version, achieved resolution, passage history, and reuse. Repeat cultures from one patient and repeated experiments with the same laboratory strain remain related observations unless independence has been demonstrated. That identity record should follow the isolate through susceptibility testing and functional assays. Such documentation keeps taxonomic claims within the achieved resolution and preserves continuity between isolate identity and subsequent corneal experiments.

### 2.3 Isolate diversity and limitations of species-level inference

Taxonomic and genotypic diversity persists even among well-authenticated ocular isolates. In a Brazilian keratitis cohort, 27 isolates comprised ten haplotypes across three species complexes, revealing substantial diversity within a single geographic region (Milanez et al., 2024). A ten-year Brazilian study resolved fusarioid isolates from keratitis and dermatomycoses across *Neocosmospora* and the *Fusarium fujikuroi* and *Fusarium oxysporum* species complexes; the collection combined clinical sites, so its pooled distribution was not specific to corneal isolates (Ferreira da Cunha Neto et al., 2024). During the U.S. contact lens-associated outbreak, 164 confirmed cases included 55 patients who underwent or were scheduled for corneal transplantation, while 39 tested isolates represented at least ten species and 19 multilocus genotypes; individual genotypes were not linked to clinical courses (Chang et al., 2006). Multilocus analysis of another outbreak collection resolved 29 haplotypes across 16 species despite a shared epidemiological source (O’Donnell et al., 2007). Earlier genotyping of 44 corneal isolates reported as *F. solani* yielded 40 fingerprints within one center, showing directly how a historical species label can conceal strain-level diversity (Godoy et al., 2004). These ocular datasets establish diversity of identity. Parallel differences in invasive behavior remain untested.

The scope of inference also depends on source composition and identification depth. In Southern China, 45 corneal isolates were analyzed together with 50 blood, skin, and other isolates, so pooled species frequencies were not cornea-specific (Guo et al., 2022). A United Kingdom reference-laboratory series grouped 234 isolates from corneal specimens or contact-lens care systems as *Fusarium* spp., precluding modern inference at the FSSC-member or haplotype level (Tuft et al., 2024). Among 30 Malaysian clinical and environmental FSSC isolates, colony and microscopic traits crossed multilocus-defined species groupings and source categories, weakening phenotype-based identification and source attribution (James et al., 2022). Morphological traits can cut across molecular groupings, and taxonomic diversity alone provides no evidence of functional divergence. Larger sample numbers do not compensate for unresolved identity or mixed provenance. Species, haplotype, source, and phenotype describe different attributes. Genotypic diversity does not establish variation in adhesion or invasion, and shared taxonomy does not guarantee equivalent corneal behavior. Mixed-source collections can characterize laboratory diversity. Inference about corneal disease requires provenance-stratified analysis and explicit reporting of identification depth. Species or species-complex identity supports evidence stratification; tissue phenotype and disease severity still require direct measurement. Direct corneal assays are needed to determine how authenticated isolates behave.

## 3 Corneal adhesion, invasion, and local dissemination

### 3.1 Adhesion and epithelial entry

Once isolate identity is established, adhesion and epithelial entry can be examined as early components of corneal pathogenicity. Experimental models begin with different biological states—intact epithelium, epithelial abrasion, contact-lens exposure, or intrastromal inoculation—and each route addresses a distinct question (Cheng et al., 2024). In an immortalized human corneal epithelial monolayer, the strain reported as *F. solani* activated focal-adhesion kinase and paxillin signaling, while genistein attenuated the adhesion-associated response; this single historical strain in a two-dimensional monolayer supports an adhesion-related host-cell pathway but provides no evidence of traversal across a multilayer barrier (Pan et al., 2011). **Table 2** summarizes ocular studies that directly connect fungal traits with invasion, host response, or tissue damage. Paired two-dimensional and three-dimensional human corneal models revealed isolate-specific differences among *F. falciforme*, *F. keratoplasticum*, and *F. petroliphilum* in adhesion, epithelial invasion, deeper tissue penetration, and host-cell damage; differential staining distinguished extracellular from internalized fungal structures, rare transcellular tunnels were observed, and early adhesion did not reliably predict later spatial phenotypes (Zimmermann et al., 2025).

**Table 2:** Ocular studies of pathogenicity and host responses relevant to FSSC keratitis.

Pathogen identity	Ocular study setting	Pathogenicity	Host response	Limitation	Reference
*F. falciforme*, *F. keratoplasticum*, *F. petroliphilum*; NRZMyk designation	hTCEpi monolayers; 3D hemi-cornea; 20 min–48 h	*F. keratoplasticum* showed the greatest invasion and damage	IL-6 and IL-8 increased without tracking invasion; TNF-α remained minimal	No recruited immunity; selected single-isolate comparisons	(Zimmermann et al., 2025)
22 FSSC outbreak isolates; multilocus genotypes	Contact-lens biofilms; murine keratitis; 48 h	FSSC formed denser biofilms; high-biofilm isolates yielded higher CFU	Corneal opacity did not track biofilm thickness	Only biofilm extremes tested *in vivo*; historical genotypes	(Mukherjee et al., 2012)
*F. solani* L-1579/2020; ITS1, ITS2	Plate biofilms; human cadaveric corneas; 24–48 h	Mature mono-species and mixed-species biofilms; MBEC exceeded planktonic MIC	No living host response was modeled	Single isolate; surface model; absent tear film	(Shivaji et al., 2023)
*F. falciforme* NRZ-2024-0725; TEF1-α, RPB2	Human trephinate; refractory disease; day 45	Diffuse full-thickness invasion with endothelial enrichment	Variable inflammatory infiltrate	Single late-stage case; unmatched comparator	(Theuersbacher et al., 2025)
Mixed fungal keratitis; genus-level culture	145-patient cohort; AqH subset; severe treated disease	*Fusarium* was the leading cultured genus	Neutrophil dominance; expanded suppressor cells; elevated IL-6 and IL-8	Mixed etiologies; selected severe cases; observational design	(Li et al., 2026)
*F. solani* AS 3.1829; collection designation	BALB/c scrape-contact-lens model; day 5	TLR9 or NLRP3 inhibition reduced corneal injury	TLR9 regulated NF-κB–NLRP3–GSDMD signaling	Single unsequenced strain; no human validation	(Zhang et al., 2026)
*F. solani* AS 3.1829; collection designation	BALB/c scrape-contact-lens model; day 5	ASO-*Osr2* improved clinical scores and histology	*Osr2* knockdown lowered IL-1β, IL-6, and TNF-α	Reused strain platform; no human validation	(Tang et al., 2025)
*F. solani*; strain not reported	C57BL/6 intrastromal model; DON-treated HCE-T cells	Infection and DON exposure produced corneal injury	PANoptosis-associated proteins and M1 signatures increased	Unverified strain; bulk inference; DON contribution unquantified	(Sha et al., 2025)
*F. solani*; hospital-prepared strain	Rabbit keratitis; DON-treated HCECs; 3–7 days	DON impaired epithelial viability, migration, and repair	Inflammatory transcripts increased	Uncharacterized strain; exogenous DON did not prove *in vivo* causality	(Shi et al., 2024)

Where sequence-based confirmation was unavailable, *F. solani* is the designation used in the source article, and member-level identity remains unresolved. Trephinate and aqueous-humor findings mainly reflect advanced, treated disease. The Zhang and Tang studies used the same strain (AS 3.1829), representing repeated testing of one isolate. 3D, three-dimensional; AqH, aqueous humor; ASO, antisense oligonucleotide; CFU, colony-forming unit; DON, deoxynivalenol; FSSC, *Fusarium solani* species complex; GSDMD, gasdermin D; HCEC, human corneal epithelial cell; HCE-T, immortalized human corneal epithelial cell line; hTCEpi, telomerase-immortalized human corneal epithelial cell line; IL, interleukin; ITS1 and ITS2, internal transcribed spacer regions 1 and 2; M1, classically activated macrophage phenotype; MBEC, minimum biofilm eradication concentration; MIC, minimum inhibitory concentration; NF-κB, nuclear factor kappa B; NLRP3, NLR family pyrin domain-containing 3; NRZMyk, German National Reference Center for Invasive Fungal Infections; RPB2, RNA polymerase II second largest subunit; TEF1-α, translation elongation factor 1-alpha; TLR9, Toll-like receptor 9; TNF-α, tumor necrosis factor alpha.

Studies of other filamentous fungi help distinguish internalization from general epithelial activation. Human corneal epithelial cells internalized *Aspergillus flavus* conidia through actin-dependent endocytosis (Arunachalam et al., 2021), and immortalized versus primary epithelial cells mounted different transcriptional responses to the same fungus (Arunachalam et al., 2022). These studies provide filamentous-fungal comparators; they do not establish FSSC entry. A contact-lens-associated murine model placed an *F. oxysporum* biofilm on an abraded epithelium and preserved a wounded surface interface, while intrastromal inoculation bypasses adhesion and epithelial traversal (Sun et al., 2010). Evidence for entry requires extracellular attachment to be distinguished from intracellular or trans-epithelial localization. Extracellular attachment, intracellular localization, trans-epithelial passage, and stromal entry should be reported as separate endpoints, together with fungal developmental phase, epithelial integrity, inoculation route, and spatial localization. Conidia associated with an injured monolayer and hyphae embedded in structured tissue represent different biological states. The three-dimensional platform cited above offers the clearest species-resolved evidence of FSSC epithelial invasion, although the frequency and route of this process in native, untreated human corneas remain unknown.

### 3.2 Stromal invasion and local dissemination

Once fungi enter the stroma, pathogenic behavior depends on how hyphae occupy and traverse the collagen-rich matrix. In lamellar keratoplasty specimens, vertical hyphal growth was associated with recurrence more often than lamella-parallel growth, although the fungal determinant responsible for this pattern was not identified (Xie et al., 2008). A late, refractory trephinate containing TEF1-α/RPB2-confirmed *Fusarium falciforme* showed diffuse full-thickness invasion with greater involvement near the endothelial surface (Theuersbacher et al., 2025). In a five-case therapeutic keratoplasty series, *Fusarium*-positive tissue was sampled after rapid progression and medical nonresponse, outside the untreated early phase (Oliver-Gutierrez et al., 2024). Recent studies using *in vivo* confocal microscopy (IVCM) and anterior-segment optical coherence tomography (AS-OCT) support *in vivo* localization and serial structural assessment, although neither method authenticates FSSC membership or establishes the viability of every visible filament (Gheorghe et al., 2025; ElZawahry et al., 2026). These observations arise from different sampling conditions. Histology records structure at excision, imaging follows morphology *in vivo*, and culture or molecular testing detects the organism.

Hyphal morphology on imaging lacks taxonomic specificity. Culture-confirmed comparisons found substantial overlap in the confocal appearance of *Fusarium* and *Aspergillus*, limiting species inference from branching patterns alone (Chidambaram et al., 2017b). Imaging syntheses and CRISPR-based testing in smear- and culture-negative keratitis supply complementary structural and organism-directed information (Cabrera-Aguas and Watson, 2023; Deivarajan et al., 2026). Both leave the isolate’s functional phenotype and viable burden across the full stromal depth unresolved. Functional tissue models interrogate fungal determinants more directly. Altering the PacC transcriptional regulator changed hyphal penetration in 27 ex vivo human donor corneas infected with *F. oxysporum*, linking this regulatory program to tissue invasion in *F. oxysporum* without extending the inference to FSSC as a whole (Hua et al., 2010). A ten-isolate chemotaxicell–scanning electron microscopy study found stronger thigmotropic responses among FSSC isolates than among isolates from the *Fusarium oxysporum* species complex, although its noncorneal platform did not examine stromal invasion in patients (Rim et al., 2026).

Reports of spatial invasion are most informative when they specify corneal compartment, depth, orientation, diffuseness, disease stage, and authentication method. Culture positivity or total fungal burden alone cannot supply this information. Cross-study comparison also requires the sampling plane, detection method, and biological meaning of a positive fungal signal. Full-thickness hyphae in therapeutic keratoplasty tissue characterize late, treated, surgically selected disease; a negative field or single time point cannot exclude spread beyond the sampled area. Deep or diffuse hyphae describe anatomy without identifying the fungal trait that produced epithelial loss, stromal melt, or visual impairment. Human evidence comes largely from *in vivo* imaging and late surgical specimens, while sequence-resolved tissue obtained early and before treatment, together with longitudinal sampling, remains scarce. Surgical specimens cannot reconstruct the route or pace of progression. Which fungal traits drive tissue damage remains unresolved.

### 3.3 Biofilm formation and toxin production

Biofilm formation is a measurable isolate phenotype, although its association with corneal injury varies with the model and endpoint. Outbreak-associated *Fusarium* keratitis isolates differed in biofilm formation, and high-biofilm isolates yielded higher corneal colony-forming unit (CFU) counts than low-biofilm isolates in a matched murine model (Mukherjee et al., 2012). Clinical and *in vitro* evidence associates fungal biofilms with reduced antifungal susceptibility and poorer treatment outcomes, although estimates vary across pathogens, biofilm assays, and clinical definitions (Utami et al., 2025). Among 29 clinical *Fusarium* isolates from several species complexes, biofilm biomass, architecture, extracellular matrix, and susceptibility varied by isolate and maturation stage (Zhang et al., 2024). A phylogenetically identified *F. falciforme* keratitis isolate formed an extracellular-matrix biofilm with a biofilm-enriched proteomic profile (Calvillo-Medina et al., 2019). Biofilm phenotypes vary at isolate level and may matter clinically; biomass alone does not measure stromal depth, corneal melt, or visual outcome.

The tissue environment further reshapes these phenotypes. On ex vivo human corneas, the strain reported as *F. solani* formed monomicrobial biofilms and mixed biofilms with ocular staphylococci, and community composition altered antimicrobial susceptibility (Shivaji et al., 2023). This model addresses polymicrobial ecology and tissue-associated growth, not a uniform monomicrobial FSSC infection. Studies of deoxynivalenol (DON) examine toxin-associated epithelial injury; they do not resolve isolate-level stromal invasion. DON was detected in *Fusarium* culture and infected rabbit cornea, and direct exposure impaired human corneal epithelial-cell viability and migration while increasing apoptosis-associated and inflammatory readouts (Shi et al., 2024). The experiment used one strain reported as *F. solani* and cannot estimate DON production across FSSC. Among 54 archived ocular isolates designated as *Fusarium*, *FUM1* was detected in 31, while genes for DON and nivalenol biosynthesis were absent (Anutarapongpan et al., 2018). The two studies measured different quantities. Biosynthetic-gene carriage does not establish toxin expression or concentration in infected tissue.

### 3.4 Fungal and host processes associated with tissue damage

Fungal and host processes can both degrade the corneal matrix. A rabbit fungal-keratitis study measured fungal enzyme activity alongside clinical and histological injury, providing direct ocular evidence of protease-associated damage in that experimental system (Gopinathan et al., 2001). Host matrix metalloproteinases also rise during infectious corneal ulceration and can sustain collagen degradation through inflammatory and nonmicrobial pathways (García-López et al., 2023). Fungal enzyme activity, host matrix remodeling, viable burden, epithelial loss, and stromal destruction are distinct outcomes. Broth assays, monolayer experiments, and late tissue specimens capture different stages of injury and should not be collapsed into a single virulence score. Biofilm, toxin exposure, fungal enzymes, and host matrix metalloproteinases have been studied in separate experiments, without matched authenticated FSSC isolates in one corneal system.

### 3.5 Isolate phenotypes and disease severity

Severity in *Fusarium* keratitis is assessed through visual acuity, infiltrate or stromal involvement, perforation, recurrence, surgery, microbiological response, and treatment failure; each endpoint captures a different aspect of disease (Cintra et al., 2024). The U.S. outbreak illustrates the unresolved relation between diversity and outcome. Of 164 confirmed patients, 55 required or were scheduled for corneal transplantation, yet the 19 multilocus genotypes identified among tested isolates were not linked to individual clinical courses (Chang et al., 2006). A sequence-resolved case attributed to *Fusarium tonkinense* combined marked antifungal resistance with destructive progression, although a single case cannot define a high-risk FSSC member (Schrecker et al., 2021). These reports show that severe disease occurs across taxonomically heterogeneous isolates without establishing a clinical severity ranking.

Clinical cohorts document patient outcomes more directly, but seldom interrogate isolate function. Among 43 molecularly identified Taiwanese cases, FSSC predominated and showed higher MICs to several agents, yet species-complex assignment was not significantly associated with corneal perforation, treatment, or final visual acuity (Huang et al., 2022b). A northwestern Spanish cohort recorded stromal involvement, corneal thinning, poor visual recovery, and surgical escalation, but included mixed fungal etiologies and did not test isolate phenotype (Lamas-Francis et al., 2024). Canadian multicenter data documented prolonged treatment and persistent visual morbidity while comparing yeast with filamentous infection, not individual FSSC members (Ling et al., 2024). A recent tertiary-referral cohort from Eastern China likewise described heterogeneous microbiology, treatment pathways, and outcomes without multilocus FSSC functional phenotyping (Yu et al., 2025). Experimental studies supply the missing functional measurements, although they rarely retain a link to longitudinal outcomes in the patients from whom the isolates were obtained.

Multilocus isolate identity, direct corneal phenotype, host-response profile, tissue drug exposure, treatment timing, and longitudinal outcome remain unmeasured within the same clinical series. Available data support pathogenic variation among isolates without establishing a reproducible species- or member-level ranking of clinical severity. Treatment escalation records failure of disease control; isolate virulence requires direct functional measurement. Severe disease is best examined by linking authenticated fungal identity and functional phenotype with host responses in the affected corneal compartment.

## 4 Corneal host defense and immunopathology

### 4.1 Epithelial sensing and regulated cell death

Fungal sensing at the corneal surface can elicit antimicrobial effector responses as well as inflammation that injures tissue. In human corneal epithelial cells challenged with the strain reported as *Fusarium solani*, Dectin-1 and TLR2 regulated antimicrobial-peptide expression without testing epithelial invasion (Kolar et al., 2017). In murine *Fusarium* keratitis, MyD88-dependent control required TLR4 and IL-1R1 but not TLR2 (Tarabishy et al., 2008). In another *F. solani* model, TLR9 silencing or inhibition of NLR family pyrin domain-containing 3 (NLRP3) reduced NF-κB/NLRP3-associated IL-1β production, stromal edema, and inflammatory infiltration, while NLRP3 activation reversed these effects (Zhang et al., 2026). Differences in cell type, inoculation method, and endpoint probably account for the receptor patterns observed; the evidence does not support a receptor hierarchy across authenticated FSSC isolates. The designs interrogate different biology. Epithelial culture captures local effector production, while murine studies incorporate stromal infection, recruited immunity, and tissue injury, so they do not constitute direct replications.

Post-transcriptional control also modifies the corneal response. Two *F. solani* studies found that METTL3 knockdown or inhibition reduced inflammatory signaling through PI3K/AKT- and NF-κB-associated axes (Huang et al., 2022a; Tang et al., 2022a). Other experiments linked miR-665-3p inhibition and miR-223-3p/ATG16L1 regulation to autophagy-associated changes in corneal inflammation (Guo et al., 2021; Tang et al., 2022b). In a comparator study of *Aspergillus fumigatus*, miR-146a-5p overexpression suppressed Dectin-1/Syk/NF-κB signaling and reduced disease-associated inflammation (Liu et al., 2026). These experiments identify regulatory mechanisms within particular systems; they do not establish the same ordering across FSSC members or across epithelial, stromal, and whole-cornea preparations. Their endpoints capture inflammation and autophagy-associated signaling, not isolate-resolved epithelial entry, restriction of viable fungus, or patient outcome.

The Nomenclature Committee on Cell Death reserves regulated cell-death labels for mechanisms supported by functional, genetic, or pharmacological evidence (Galluzzi et al., 2018). Oxidative-stress experiments supported RIPK3/MLKL-dependent corneal epithelial necroptosis together with NLRP3 signaling (Wang et al., 2023a). In *A. fumigatus* keratitis, perturbation of the reactive oxygen species/caspase-3/GSDME axis supported epithelial pyroptosis as a comparator mechanism (Kan et al., 2025). *Fusarium solani* infection and DON exposure produced coordinated changes in caspases, gasdermins, and MLKL, together with terminal deoxynucleotidyl transferase dUTP nick-end labeling and transcriptomic signatures, without functional blockade of the core PANoptosis machinery (Sha et al., 2025). Without such blockade, the results support PANoptosis-associated signatures but do not establish a complete causal death program. Time-course transcriptomics further showed that death-related programs shift during fungal-keratitis progression (Xu et al., 2023). Epithelial sensing, autophagy, and regulated death should be analyzed separately, with cell type, organism, infection stage, and perturbation specified. Assigning a whole-cornea protein or transcript change to epithelium, stromal cells, or infiltrating leukocytes requires direct localization of the responsible cell population.

### 4.2 Stromal damage and repair

Stromal injury encompasses several concurrent processes. Infectious corneal ulcers involve matrix metalloproteinase-mediated collagen degradation from epithelial cells, keratocytes, infiltrating leukocytes, and other host compartments, while spatial multi-omics in fungal keratitis has identified altered sphingolipid homeostasis across infected tissue (García-López et al., 2023; Fu et al., 2025). In rabbit keratitis caused by the strain reported as *F. solani*, infection increased inflammatory and oxidative-stress readouts in corneal tissue without isolating a single causal stromal pathway (Ruban et al., 2018). Viable fungal burden, oxidative stress, spatial lipid state, collagen loss, opacity, and tissue architecture measure different features of disease. Their trajectories may diverge. Late opacity can reflect persistent organisms, inflammatory proteolysis, keratocyte loss, collagen disorganization, and fibrotic remodeling in different proportions. A lower clinical score at one time point cannot identify which stromal component has been preserved or restored. Neutrophil recruitment is essential for fungal control, but excessive or prolonged neutrophilic inflammation may also aggravate stromal destruction through degranulation, proteolytic activity, and oxidative injury (Abbondante et al., 2023; García-López et al., 2023). Neutrophil infiltration should therefore be interpreted together with viable fungal burden and stromal integrity.

Toxins may initiate epithelial injury before wider stromal damage develops. DON was detected in *Fusarium* culture and infected rabbit cornea, and direct exposure impaired human corneal epithelial-cell viability and migration while increasing apoptosis-associated and inflammatory readouts (Shi et al., 2024). Transcriptomic analysis of purified toxin identified exposure-specific immune and metabolic responses in the absence of live infection; it defines the consequences of toxin exposure, not toxin production or stromal penetration during keratitis (Sha et al., 2026). Host perturbation experiments reveal the same dissociation between tissue injury and pathogen control. Hypoxia-inducible factor 1-alpha (HIF-1α) inhibition in *A. fumigatus* keratitis improved epithelial repair and inflammatory readouts while fungal burden increased (Yang et al., 2025a). Tissue protection can improve despite poorer fungal control, and this experiment does not constitute a treatment recommendation. Measures of epithelial viability or cytokine reduction should be accompanied by direct assessment of collagen integrity, stromal cell survival, and fungal viability.

Repair and acute inflammation unfold on different trajectories. IL-24 manipulation linked caspase-11/GSDMD signaling to corneal inflammation and fibrosis in experimental fungal keratitis (Yang et al., 2026). Stromal repair more broadly depends on keratocyte survival, myofibroblast differentiation, basement-membrane integrity, collagen and proteoglycan organization, and biomechanical cues (Mohan et al., 2022). Recovery should be assessed through pathogen control, matrix preservation, epithelial closure, keratocyte viability, fibrosis, and transparency at defined times and treatment exposures. Existing repair studies examine individual strains in specific models, leaving cross-isolate differences in repair and scarring unresolved. Longitudinal studies should distinguish early loss of transparency, persistent myofibroblasts, and later collagen reorganization instead of reducing these processes to a terminal opacity score.

### 4.3 Neutrophil and macrophage responses

Myeloid recruitment can constrain fungal growth and amplify corneal injury. In a selected aqueous-humor and peripheral-blood subset of a 145-patient fungal-keratitis cohort, immune profiling identified a neutrophil-dominant ocular response, although mixed etiologies and selected sampling limit inference about untreated FSSC infection (Li et al., 2026). Murine *Aspergillus* and *Fusarium* infection models showed that IL-17-producing neutrophils and Th17 cells regulate fungal growth and corneal disease (Taylor et al., 2014). Recent *F. solani* mouse work further found that vitamin C enhanced neutrophil antifungal activity while altering inflammatory and corneal readouts (Xie et al., 2026). Neutrophil abundance or myeloperoxidase activity alone cannot establish whether the net effect is protective. Viable burden, degranulation or oxidative injury, stromal architecture, and sampling time are needed to interpret that contribution.

Macrophages influence disease through recruitment, phagocytic control, and inflammatory activation. In an *A. fumigatus* comparator, MYO1F knockdown reduced myeloid recruitment, MyD88/NF-κB signaling, cytokine production, and corneal inflammation (Liu et al., 2024). Direct *Fusarium* work showed that experimentally activated macrophages intensified corneal inflammation despite low recoverable colony counts, consistent with an injury-amplifying state in the strain reported as *F. solani* (Hu et al., 2014). Three *A. fumigatus* comparator studies used LC3-associated phagocytosis, Piezo1, or IL-36γ to connect autophagic flux, calcium signaling, inflammasome activity, phagocytosis, and fungal control (Luan et al., 2024; Yang et al., 2025b; Niu et al., 2026). These pathways provide testable hypotheses for macrophage function in FSSC keratitis. Direct evidence is still needed before they can be generalized as an FSSC-wide receptor, cytokine, or autophagy program.

Direct *F. solani* studies have also revealed metabolic and death-related macrophage states. PFKFB3 and glycolytic flux increased in infected corneas and bone-marrow-derived macrophages; PFKFB3 depletion raised murine corneal clinical scores, stromal infiltration, and PI3K/AKT/NF-κB-associated inflammation without establishing a corresponding change in fungal burden (Zhang et al., 2025b). Targeted ZBP1 perturbation connected Z-form nucleic acids with macrophage PANoptosis, fungal immune escape, extracellular DNA, and biofilm behavior within one *F. solani* cell-and-mouse platform (Gao et al., 2026). Recruitment, phagocytosis, inflammatory output, metabolism, and regulated death are distinct macrophage functions in these studies. Polarization markers become more informative when interpreted alongside viable fungal burden, phagocytic function, and corneal injury. Direct *Fusarium* evidence remains concentrated in a small number of historical strains and laboratory platforms. Comparisons among isolates will be stronger when the same strain is followed through macrophage perturbation, fungal-burden assessment, and tissue-damage analysis. Cell origin and sampling time also matter. Bone-marrow-derived macrophages, resident corneal myeloid cells, and newly recruited cells contribute differently to inflammatory and phagocytic readouts. Changes in polarization markers, cytokine concentrations, or corneal scores alone cannot establish improved fungal control.

### 4.4 Fungal clearance and collateral tissue damage

Fungal clearance and corneal preservation can follow separate trajectories. Human corneal tissue infected with isolates reported as *F. solani* or *A. flavus* expressed shared innate and adaptive immune mediators, showing that late surgical specimens contain broad responses to filamentous fungi that cannot be assigned to FSSC identity alone (Karthikeyan et al., 2011). Such specimens connect experimental observations with human disease, but surgical selection and prior treatment prevent reconstruction of the early sequence from sensing to clearance. Culture status, tissue architecture, opacity, and visual outcome reflect different processes and time points. Negative smear or culture results do not exclude fungal infection: tear β-D-glucan can provide a rapid, non-species-specific diagnostic signal, while organism-directed molecular testing may remain positive in conventionally negative cases (Tawde et al., 2024; Deivarajan et al., 2026). A negative surface culture cannot confirm clearance from deeper tissue. Persistent opacity does not prove viable fungus, and early improvement in inflammation does not demonstrate eradication unless viability is assessed concurrently. Corneal and anterior-chamber specimens sample distinct ocular compartments and may yield different results.

Mechanistic studies illustrate several relations between fungal killing and host injury. In *A. fumigatus* keratitis, cathelicidin enhanced neutrophil phagocytosis and intracellular conidial degradation, supporting a cell-mediated clearance mechanism (Hou et al., 2024). Integrated proteomic and metabolomic analysis linked purine-metabolism changes in infected mouse and human corneas with *in vitro* inhibition of *F. solani* by adenosine and adenosine monophosphate, without establishing exposure in infected tissue or clinical efficacy (Zhang et al., 2025a). In a mouse fungal-keratitis model treated with ebselen and an *A. fumigatus* PIM1-inhibition model, fungal burden changed alongside inflammatory or oxidative-injury readouts (Yu et al., 2024; Han et al., 2026). Uniform activity across FSSC and improvement in human vision were not tested. Within each experimental system, however, a single intervention altered fungal burden and host-damage measures together; conclusions remain limited to the pathogen, exposure compartment, and outcome studied.

Ocular antifungal immunity depends on epithelial, stromal, myeloid, and soluble-effector compartments whose protective and injurious contributions vary with organism, tissue location, and disease stage (Abbondante et al., 2023). Clearance requires a viable-burden measure, tissue protection requires spatial injury, matrix, repair, or transparency measures, and clinical benefit requires patient-level outcomes. Inflammatory markers alone do not demonstrate antifungal activity; lower culture counts without matrix or repair assessment do not establish tissue protection. The most informative designs measure viable burden, spatial fungal localization, inflammatory injury, matrix integrity, epithelial closure, and transparency at matched times using the same authenticated isolate. Culture, quantitative molecular assays, histology, and imaging can all contribute, although each samples viability, depth, and spatial coverage differently. Human visual benefit cannot be inferred from an animal corneal score. Separating these outcomes permits host-defense effects to be evaluated independently of treatment development and provides criteria for comparing cell, tissue, animal, and human-specimen models. **Figure 1** summarizes the relationships among fungal viability, host-response state, tissue injury, and repair.

**Figure 1:**
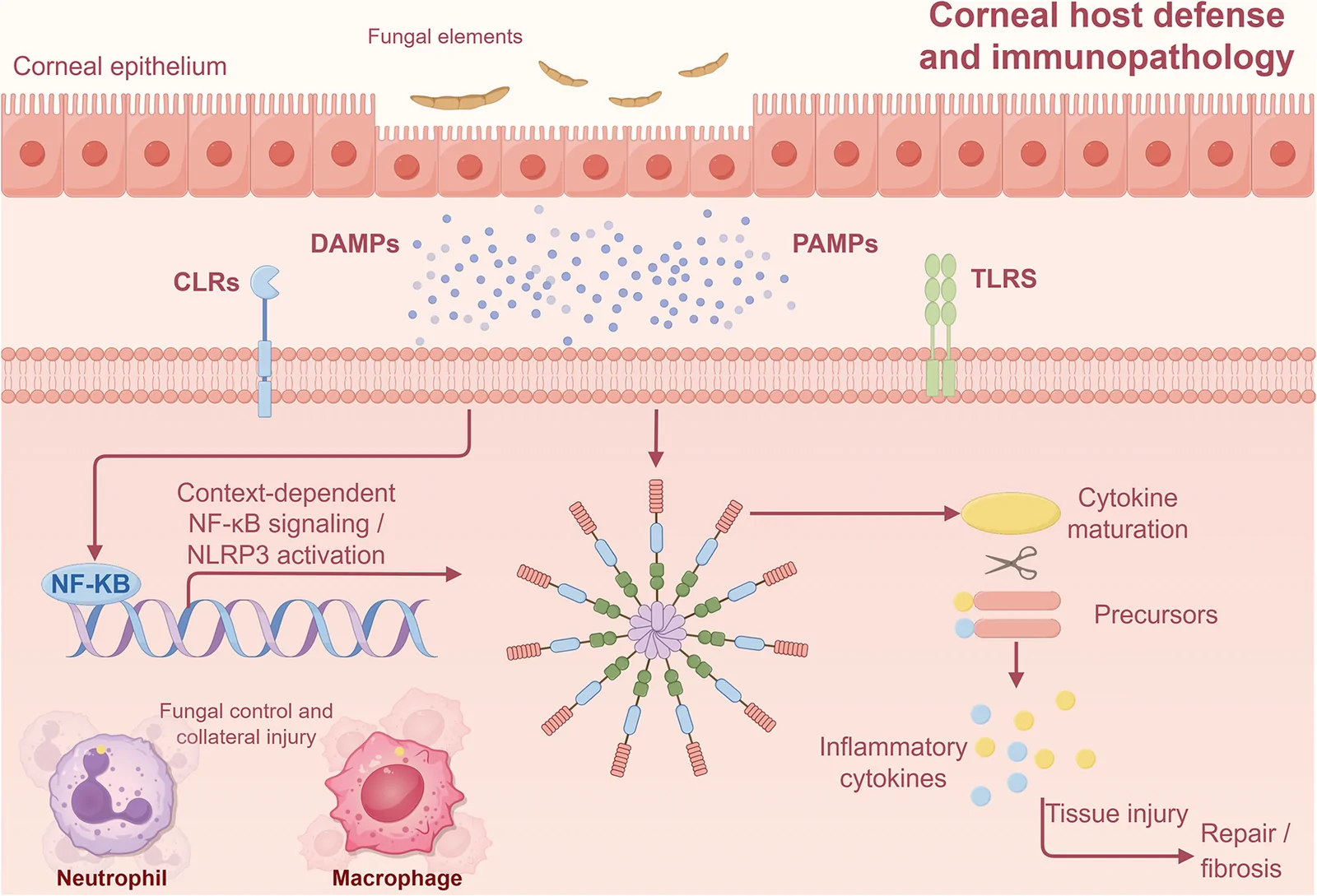
Corneal host defense and immunopathology in FSSC keratitis. *Fusarium*-like fungal elements at a disrupted corneal epithelial surface provide pathogen-associated molecular pattern signals, whereas injured host cells release damage-associated molecular patterns. These signals are sensed through pattern-recognition receptors, including C-type lectin receptors and Toll-like receptors, and may engage context-dependent NF-κB signaling and NLRP3 activation. The central radial complex represents the NLRP3 inflammasome, and the NF-κB-to-NLRP3 arrow denotes a context-dependent priming relationship rather than a universal linear pathway. NLRP3-associated cytokine maturation and release, exemplified by IL-1β-associated signaling, can amplify corneal tissue injury. Neutrophils and macrophages may contribute to fungal control while also promoting collateral injury; therefore, fungal clearance and tissue preservation can diverge. Subsequent outcomes include epithelial and stromal repair or fibrotic remodeling. The scheme is conceptual and does not imply a fixed receptor hierarchy, uniform pathway ordering across FSSC isolates, or assignment of all depicted events to a single cell type. CLRs, C-type lectin receptors; DAMPs, damage-associated molecular patterns; FSSC, *Fusarium solani* species complex; IL-1β, interleukin-1 beta; NF-κB, nuclear factor kappa B; NLRP3, NLR family pyrin domain-containing 3; PAMPs, pathogen-associated molecular patterns; TLRs, Toll-like receptors.

## 5 Experimental models of FSSC keratitis

### 5.1 Cell-based systems

Cell-based systems enable precise causal analysis within defined host compartments, with tissue architecture left unmodeled. Human corneal epithelial monolayers have supported controlled perturbation of receptor-dependent antimicrobial output and regulated-death machinery (Kolar et al., 2017; Wang et al., 2023a). Corneal cell cultures have likewise enabled targeted studies of METTL3-associated inflammatory signaling and miR-223-3p/ATG16L1-linked autophagy (Huang et al., 2022a; Tang et al., 2022b). Genes, inhibitors, exposure time, and cell source can be controlled, and intracellular or secreted readouts collected over short intervals. Monolayer association, colony recovery, or transcriptional activation alone cannot distinguish extracellular attachment, internalization, barrier crossing, and stromal invasion. Macrophage and epithelial–macrophage cocultures can examine directional cell–cell communication, phagocytosis, metabolism, or death programs; they omit multilayered epithelium, collagen architecture, resident cell diversity, vascular recruitment, tear dynamics, and tissue-level drug gradients. Interpretation depends on the reported cell source, fungal developmental phase, multiplicity of infection, exposure duration, localization method, and viability readout. Cell systems are well suited to causal questions within one compartment. Spatial invasion, multicellular injury, recruited immunity, and treatment penetration require more complex models. Immortalized and primary cells should be distinguished by source and differentiation state; technical wells or fields from one culture should be analyzed as within-experiment replicates, and strain comparisons should retain authentication and passage history.

### 5.2 Tissue-based corneal models

Structured human-derived models introduce epithelial layering, stromal matrix, or both. A paired two-dimensional epithelial and three-dimensional human hemi-cornea platform compared authenticated *F. falciforme*, *F. keratoplasticum*, and *F. petroliphilum* isolates while retaining a multilayered epithelium over a stromal compartment; adhesion, internalization, tissue penetration, host-cell damage, cytokine release, and spatial localization could all be examined within one model family (Zimmermann et al., 2025). A primary human fibroblast-derived construct and an ex vivo goat corneal model also preserved stromal features during *Fusarium* infection, although neither recruited circulating cells. The former resolved *F. petroliphilum* penetration, collagen disruption, host cytokine and metalloproteinase responses, and fungal transcriptional adaptation; the latter enabled tissue immune and cytokine measurements together with antifungal testing (Brown et al., 2021b; Trivedi et al., 2025). These systems add complexity in different ways and leave different gaps. The hemi-cornea preserves an epithelial–stromal interface without recruited circulation, the fibroblast-derived matrix isolates stromal occupation without a surface barrier, and the goat cornea retains organ-level tissue without systemic recruitment. Labels such as three-dimensional or ex vivo do not imply equivalent biological fidelity.

Ex vivo corneas retain lamellar collagen, resident corneal cells, and physical gradients absent from monolayers, although they generally lack circulation, systemic physiology, and newly recruited leukocytes (Cheng et al., 2024). Paired rabbit and human ex vivo corneas supported *F. solani* infection after surface scarification or intrastromal injection, with CFU recovery and histology used to compare route- and tissue-dependent infection patterns (Pinnock et al., 2017). Eye-banked human corneas infected with *F. oxysporum* allowed confocal quantification of hyphal volume after photoactivated cross-linking (Alshehri et al., 2016). A porcine model linked fungal morphology and structural change with antifungal response after infection by the strain reported as *F. solani* (Miyakubo et al., 2026), while human cadaveric corneas supported monomicrobial and mixed biofilms with ocular staphylococci (Shivaji et al., 2023). These platforms reproduce tissue occupation, local exposure, or polymicrobial growth. They do not reproduce systemic immunity, human pharmacokinetics, or patient outcomes. Their principal strength is concurrent visualization of fungal location and tissue architecture; a reduction in signal still requires a viability measure before it can be interpreted as killing.

Model construction determines which tissue process becomes visible. Epithelial integrity, tissue orientation, wound geometry, inoculum placement, culture duration, oxygenation, medium movement, and drug-delivery direction can each shift the dominant readout. A human cornea-on-a-chip reproduced a multilayered epithelial–fibroblast barrier after bacterial infection, providing an architectural and methodological precedent without direct FSSC validation (Deng et al., 2024). Recent cornea-chip synthesis likewise identifies flow, barrier composition, cell selection, and mechanical forces as active determinants of the model phenotype (Kim et al., 2025). Multiple tissue pieces from one donor should be treated as within-donor replicates, with donor number reported separately from sample number. This prevents subdivision of a single cornea among treatment groups from inflating the count of independent biological samples.

### 5.3 Animal keratitis models

Animal keratitis models add tear exposure, recruited immunity, serial examination, and ocular dosing; inoculation route and host manipulation determine the biological question. Intrastromal murine infection with *Fusarium* tested requirements for MyD88, TLR4, and IL-1R1 after fungi were placed directly in the stroma (Tarabishy et al., 2008). A contact-lens-associated model placed an *F. oxysporum* biofilm on abraded epithelium, preserving a wounded surface interface through which barrier injury, lens exposure, fungal growth, and innate immune deficiency could be followed (Sun et al., 2010). One design begins with established stromal infection, the other with epithelial-surface exposure. Both sample limited segments of the natural history. Immunodeficiency or pharmacological pretreatment can clarify pathway dependence, but it also changes disease kinetics relative to immunocompetent infection. Inoculum size, fungal developmental state, epithelial trauma, and the interval from inoculation to treatment further determine whether the model represents initial entry, established stromal infection, or refractory disease.

Animal systems permit longitudinal measurement of fungal and host outcomes in the same eye. Murine genetic tools support causal perturbation, whereas the larger rabbit cornea facilitates repeated examination and topical dosing. Human, murine, and rabbit corneas differ substantially in diameter, thickness, and anterior stromal organization, and the mouse cornea also shows stromal architecture that differs from that of the human cornea (Henriksson et al., 2009). A distinct Bowman’s layer is present in the human cornea, is absent in adult rabbits, and has not been detected in commonly used C57BL/6 and BALB/c mouse strains; however, its role as a physical barrier remains uncertain (Wilson, 2020). These interspecies differences may influence the relative depth of inoculation, hyphal penetration, and local drug distribution and should therefore be considered when extrapolating animal findings to human keratitis. In one *A. fumigatus* experiment, HIF-1α inhibition improved inflammatory and repair readouts while fungal burden increased, demonstrating that tissue injury and clearance can diverge in an animal model without assigning the pathway to FSSC (Yang et al., 2025a). Rabbit models using strains reported as *F. solani* enabled serial clinical scoring, histology, and viable-burden assessment during delayed topical therapy, comparing natamycin, voriconazole, and amphotericin B in an immunosuppressed model and luliconazole with natamycin in a separate study (Aung et al., 2020; Arimoto et al., 2023). These experiments captured delayed-treatment kinetics but did not measure human stromal pharmacokinetics or visual function. Animal clinical scores should be interpreted alongside viable burden and histology at matched times, with host species, immune state, trauma, inoculum, route, and treatment timing specified. Opacity, epithelial defects, fibrosis, perforation, and visual function are distinct disease features, and individual models may reproduce them with different fidelity.

### 5.4 Human corneal specimens

Human corneal specimens provide the closest link to disease, although most are cross-sectional, obtained after treatment, and selected through a clinical procedure. Formalin-fixed corneas analyzed by 3′ RNA sequencing using massive analysis of cDNA ends distinguished herpes simplex virus from a combined bacterial/fungal response group, but the latter contained only one specimen with fungal keratitis (Lapp et al., 2024). Therapeutic keratoplasty corneas attributed to *Fusarium* or *A. flavus* showed shared and pathogen-associated inflammatory programs in advanced, post-treatment disease without isolate-level FSSC resolution (Dinesh et al., 2025). Pooled fungal-keratitis corneas yielded a wound- and inflammation-associated microRNA profile without species or isolate resolution (Boomiraj et al., 2015), and separate late-stage transcriptomics identified persistent innate and extracellular-matrix programs in surgically selected microbial keratitis (Chidambaram et al., 2017a). Bulk profiles reveal programs present in diseased tissue while combining signals from resident corneal cells and infiltrating leukocytes, with no spatial assignment. They can reveal pathways operating in human cornea but cannot identify the responsible cell type, reconstruct temporal sequence, or demonstrate dependence on a particular isolate trait.

Histology supplies the spatial information absent from bulk molecular profiles. Human corneas infected with isolates reported as *F. solani* or *A. flavus* showed overlapping expression of innate and adaptive immune mediators (Karthikeyan et al., 2011). One late emergency keratoplasty trephinate contained TEF1-α/RPB2-confirmed *F. falciforme* with diffuse full-thickness invasion, although the evidence derives from a single FSSC case (Theuersbacher et al., 2025). Lamellar keratoplasty specimens distinguished horizontal from vertical hyphal growth, and vertical growth was associated with recurrence without revealing a fungal mechanism (Xie et al., 2008). A five-case *Fusarium* series likewise sampled rapidly progressive infection after medical nonresponse (Oliver-Gutierrez et al., 2024). In a culture-proven fungal-keratitis series, anterior-chamber exudate culture, corneal-button culture, and histology yielded different results, showing that compartments and assays capture different aspects of infection (Kaur et al., 2025). Human specimens provide direct evidence of spatial invasion and host response in diseased corneas. Their interpretation depends on surgical selection, prior treatment, identification resolution, sampled compartment, and timing. Surgical tissue captures a single late time point and cannot reconstruct untreated early disease or within-patient progression.

### 5.5 Concordance across models

Cross-model agreement can be tested within one study or inferred more cautiously across separate studies. The strongest within-study comparisons carry the same authenticated isolate across models and prespecify differences in developmental phase, inoculum, treatment, sampling time, and readout. For *F. keratoplasticum*, azole susceptibility differed among conidia, hyphae, and porcine corneal infection, directly showing that a conidial assay need not reproduce hyphal or tissue response (Roberts et al., 2023). Carrying the isolate forward reveals whether a phenotype persists or changes as fungal state and biological complexity vary. Comparisons based on unrelated isolates or endpoints cannot resolve that question. Directional agreement has limited evidentiary value when fungal state or measurement differs between model layers.

Across independent studies, model choice can alter the phenotype under observation and weaken apparent agreement. Reduced *F. oxysporum* hyphal volume in an ex vivo cross-linking system demonstrated a tissue response without recruited immunity, ocular pharmacokinetics, or patient outcomes (Alshehri et al., 2016). A cornea-on-a-chip resolved epithelial, fibroblast, barrier, and intervention effects, but infection was validated with bacteria (Deng et al., 2024). Late-stage human transcriptomics sampled surgically selected disease (Chidambaram et al., 2017a), and patient confocal microscopy showed overlapping morphologic appearances of *Fusarium* and *Aspergillus* (Chidambaram et al., 2017b). Similar changes in burden, inflammation, or structure amount to validation only when organism, compartment, stage, and endpoint are comparable. Shared terminology can create an impression of concordance while the underlying studies examine different biological processes.

Available studies do not provide a continuous matched-isolate chain from epithelial culture and structured human corneal tissue to an immune-competent animal and paired patient material with harmonized endpoints. Concordance should be assessed separately for identity, adhesion, stromal depth, viable burden, host-cell injury, immune-cell state, drug exposure, tissue preservation, and clinical outcome. What remains missing is a matched isolate–model–patient chain that identifies which observations reproduce across systems, which depend on model context, and which help explain treatment failure.

The primary studies used different histological methods. Fixation, embedding, sectioning, staining, and the criteria used to assess fungal invasion or host tissue injury were reported in varying detail, limiting direct comparison of histological findings. Reports also differed in their documentation of isolate authentication, culture purity, blinded assessment, biological and technical replication, statistical analysis, and controls used during imaging or quantification across experimental batches. Future experimental studies should describe these procedures and controls explicitly. **Figure 2** depicts these dimensions of fidelity and the matched-isolate validation sequence, while **Table 3** compares the complementary capabilities and limitations of the main model systems.

**Figure 2:**
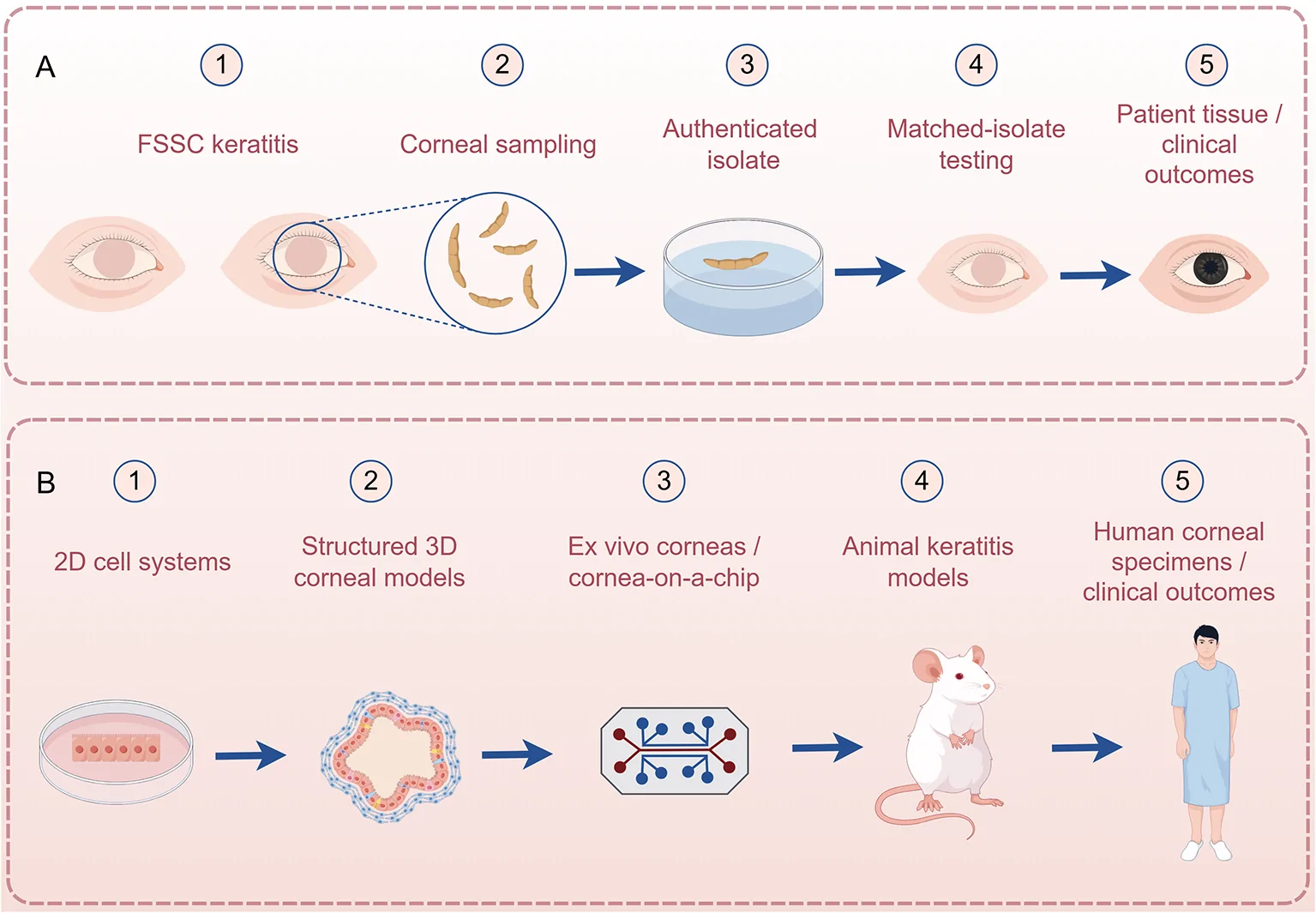
Proposed matched-isolate validation sequence and domain-specific model fidelity in FSSC keratitis. **(A)** A proposed matched-isolate sequence begins with FSSC keratitis and corneal sampling, followed by recovery and taxonomic authentication of the clinical isolate, testing of the same authenticated isolate in experimental systems, and analytical linkage of model findings to patient tissue and clinical outcomes. The culture dish is schematic; isolate authentication requires a traceable identity record and taxonomic confirmation appropriate to the resolution claimed, rather than culture recovery alone. The arrow from matched-isolate testing to patient tissue and clinical outcomes denotes concordance assessment and analytical linkage, not a causal effect of model testing on patient outcomes. A continuous isolate–model–patient chain with harmonized endpoints has not yet been achieved. The depicted fungal elements are schematic, and conidial and hyphal states represent biologically distinct developmental phases. **(B)** Complementary evidence is provided by 2D cell systems, structured 3D corneal models, ex vivo corneas and cornea-on-a-chip, animal keratitis models, and human corneal specimens and clinical outcomes. The arrows indicate increasing tissue, host, and clinical context rather than a mandatory experimental sequence, direct translational equivalence, or overall model superiority. Model fidelity is domain-specific because each system reproduces selected features of FSSC keratitis while omitting others. Human corneal specimens provide direct evidence of disease but are commonly cross-sectional, treatment-exposed, and selected by clinical procedures. Cornea-on-a-chip represents an architectural and methodological precedent from bacterial keratitis and has not been directly validated for FSSC keratitis. 2D, two-dimensional; 3D, three-dimensional; FSSC, *Fusarium solani* species complex.

**Table 3:** Experimental models of FSSC and *Fusarium keratitis*: disease features, readouts, and limitations.

Pathogen	Model system	Infection design	Reproduced disease features	Principal readouts	Model limitation	Reference
Clinical *F. petroliphilum* isolate	Human 3D stromal construct	5 × 10^4^ conidia; 24–48 h	Hyphal invasion; ECM damage; fibroblast inflammation	Histology; TEM; RNA-seq; cytokines	No epithelium, recruited immunity, nerves, or tear film; one isolate	(Brown et al., 2021b)
*F. solani* NCPF 2699	Ex vivo rabbit and human corneas	Scarification or intrastromal inoculation; 24–48 h	Haze; surface growth; stromal penetration; mixed infection	CFU; PAS; fluorescence imaging	Very high inoculum; absent recruited immunity and tear film	(Pinnock et al., 2017)
*F. solani* CSH_1	Ex vivo caprine cornea	10^4^ spores; 6 days; treatment after 48 h	Hyphal invasion; tissue injury; resident myeloid activation	H&E; flow cytometry; ELISA	No systemic immunity or pharmacology; one unsequenced isolate	(Trivedi et al., 2025)
FSSC5 LSEM 4652	Ex vivo porcine cornea	10^5^ conidia; treatment on days 2–3	Midstromal invasion; drug-specific hyphal changes	Microscopy; qPCR; CFU; PAS	No immunity or tear dynamics; one strain and dose	(Miyakubo et al., 2026)
*F. keratoplasticum* FK1, haplotype 2u	Paired *in vitro* and porcine ex vivo models	Azoles at 0, 24, and 48 h	Germination; stage-dependent susceptibility loss	Time-lapse imaging; absorbance; growth area	One equine isolate; n = 3; no viability, histology, or immunity	(Roberts et al., 2023)
FOSC MRL8996	Contact-lens biofilm mouse model	Biofilm lens on abraded wild-type and immune-deficient corneas	Rapid opacity; neutrophil clearance; deep invasion under immune deficiency	Opacity; CFU; PAS; cytokines	FOSC; reused isolate; short disease course	(Sun et al., 2010)
*F. solani* ATCC 46492	Immunosuppressed rabbit model	Midstromal injection; 12 days; treatment from day 5	Ulceration; hypopyon; neovascularization; anterior-chamber invasion	Slit lamp; ulcer area; H&E; GMS; CFU	Artificial entry; profound immunosuppression; n = 4	(Aung et al., 2020)

The models differ in tissue architecture, immune recruitment, inoculation route, and treatment exposure. Taxonomic names follow the original studies, and unsequenced *F. solani* strains remain unresolved at the FSSC-member level. Drug responses observed in animal and ex vivo systems require confirmation in clinical studies. 3D, three-dimensional; ATCC, American Type Culture Collection; CFU, colony-forming unit; ECM, extracellular matrix; ELISA, enzyme-linked immunosorbent assay; FOSC, *Fusarium oxysporum* species complex; FSSC, *Fusarium solani* species complex; FSSC5, *Fusarium solani* species complex phylogenetic species 5; GMS, Grocott–Gomori methenamine silver stain; H&E, hematoxylin and eosin; NCPF, National Collection of Pathogenic Fungi; PAS, periodic acid–Schiff stain; qPCR, quantitative polymerase chain reaction; RNA-seq, RNA sequencing; TEM, transmission electron microscopy.

## 6 Antifungal response and refractory infection

### 6.1 Antifungal susceptibility, tolerance, and corneal tissue drug exposure

The meaning of an MIC depends on the fungal state tested, the resistance mechanism, the exposure compartment, and the clinical endpoint. A United Kingdom reference-laboratory series found *Fusarium* common among ocular molds and generally more susceptible *in vitro* to natamycin than to voriconazole, although most isolates were reported only at genus level (Tuft et al., 2024). A molecularly characterized Brazilian cohort of corneal isolates showed heterogeneous susceptibility while retaining species-complex identity (Milanez et al., 2024). A Mexican clinical-isolate series composed largely of fusariosis cases reported nonocular comparative susceptibility data after TEF1-α identification (Román-Montes et al., 2025). A South Indian *post hoc* analysis found rising natamycin MICs over time, including among *Fusarium* isolates, but identified no resistance mechanism or link to individual clinical courses (Prajna et al., 2022). Nonocular three-locus data likewise showed intra- and interspecies variation in olorofim MICs (Xue et al., 2025). Ocular studies have not yet validated a defined resistance determinant and linked it to both MIC and outcome in the same patients with FSSC keratitis. Genetic resistance, planktonic susceptibility, and clinical failure are related but non-equivalent components of antifungal response.

Planktonic MICs and clinical treatment effects answer different questions. Across isolates from the first Mycotic Ulcer Treatment Trial and a recent clinical resistance–outcome study, higher MICs or resistant phenotypes were associated with selected adverse outcomes, although the associations varied by endpoint (Sun et al., 2014; Vishwakarma et al., 2025). Recent treatment syntheses favor topical natamycin over topical voriconazole for filamentous and *Fusarium* keratitis and emphasize the limited evidence for other regimens (Hoffman et al., 2022; Farnan et al., 2026). In a later randomized study in which both groups also received oral ketoconazole, adding topical voriconazole to natamycin did not improve healing or vision over natamycin alone (Di Zazzo et al., 2025). The later *Fusarium*-specific secondary analysis of the second Mycotic Ulcer Treatment Trial yielded an exploratory subgroup signal, while treatment-strategy syntheses retain the negative overall trial result (Prajna et al., 2017; Storr et al., 2024). Neither evidence base measured stromal drug exposure or hyphal depth. Estimates remain specific to the regimen and endpoint studied. An MIC or a change in administration route alone cannot demonstrate effective stromal exposure or patient benefit.

Phenotypic tolerance and validated genetic resistance arise through different processes. Outbreak-associated *Fusarium* keratitis isolates differed in biofilm formation, and biofilm growth reduced susceptibility relative to planktonic growth (Mukherjee et al., 2012). Across 29 clinical *Fusarium* isolates from several species complexes, biomass, architecture, extracellular matrix, and susceptibility varied by isolate and biofilm maturation stage (Zhang et al., 2024). For authenticated *Fusarium keratoplasticum*, azole activity differed among conidia, hyphae, and ex vivo corneal infection, showing that a conidial MIC may poorly represent the fungal phase present in tissue (Roberts et al., 2023). A porcine ex vivo model also connected fungal-element morphology with tissue-level responses to natamycin, voriconazole, and luliconazole, although it remained a controlled exposure system and did not model patient response (Miyakubo et al., 2026). Conidial MIC, hyphal susceptibility, biofilm tolerance, biofilm maturation, and tissue-phase response should be reported as separate resistance-related measures.

Drug exposure imposes a separate pharmacological constraint. In therapeutic keratoplasty specimens, intracorneal natamycin or voriconazole concentrations did not consistently align with MIC_90_ values or clinical response, although the samples represented late, treated, surgically selected disease (Patil et al., 2025). Voriconazole measurements in aqueous humor establish exposure in that fluid after topical or combined dosing; depth-resolved concentrations across infected stroma were not measured (Thiel et al., 2007). Reviews of natamycin delivery and recent nanoparticle, microneedle, and collagen-binding platforms identify suspension behavior, solubility, corneal residence, and formulation-dependent penetration as determinants of local exposure (Mascarenhas et al., 2023; Cai et al., 2025; Hu et al., 2025; Khotcharrat et al., 2025). A 2026 bilayer dissolving microneedle system improved controlled ocular delivery of voriconazole but did not establish exposure in infected human stroma or patient efficacy (Gowda et al., 2026). A mechanistic account of refractory infection should retain planktonic susceptibility, defined resistance determinants, biofilm or hyphal tolerance, viable tissue burden, corneal and aqueous exposure, and clinical outcome as separate measurements.

### 6.2 Host-directed and combination treatment strategies

Adjunctive approaches seek to limit host injury, target the fungus directly, or enlist immune effectors. Antisense inhibition of the long non-coding RNA *Osr2* reduced *Xcr1*-linked inflammatory progression in experimental keratitis caused by the strain reported as *Fusarium solani*, without demonstrating direct fungal killing (Tang et al., 2025). Targeted ZBP1 perturbation connected macrophage PANoptosis, extracellular nucleic acids, immune escape, and biofilm behavior within one *F. solani* cell-and-mouse platform, identifying a mechanistic candidate that has not been validated as treatment (Gao et al., 2026). S100A12 directly disrupted *Fusarium* membranes and inhibited growth and biofilm formation (Roy et al., 2024). SA-XV was tested against *Fusarium* spp. without member-level FSSC authentication and reduced fungal growth, biofilm formation, and murine corneal burden (Agarwal et al., 2025). Manogepix was evaluated against eight clinical keratitis isolates reported as *F. solani* and in a rabbit *F. solani* keratitis model (Gowtham et al., 2026). Neither the SA-XV nor the manogepix study established patient efficacy. A corneal-targeting peptide active against *F. dimerum* provides a non-FSSC *Fusarium* comparator (Arora et al., 2025). An antibody against poly-N-acetylglucosamine reduced fungal burden and corneal pathology through host immune components, consistent with an immune-effector-dependent adjunct rather than direct drug action (Zhao et al., 2016).

Combination studies should separate *in vitro* interaction, ocular exposure, tissue preservation, and clinical effect. Chlorhexidine combinations were tested against 194 corneal *Fusarium* isolates, while the accompanying small patient series remained exploratory and could not establish comparative efficacy (Kang et al., 2025). Atovaquone and propranolol showed experimental activity against *Fusarium* corneal isolates, but animal validation was absent or confined to an *Aspergillus* model (Clark et al., 2018; Zegans et al., 2025). Two *A. fumigatus* studies combined antifungal and inflammation-modulating effects through HMGB1/LOX-1 or autophagy/Nrf2 pathways (Cong et al., 2024; Yu et al., 2026). These filamentous-fungal comparators illustrate the value of measuring fungal and host outcomes together. They provide no direct evidence of an FSSC treatment effect.

Clinical studies ultimately determine whether these approaches improve patient outcomes. A 34-eye pilot trial of topical caspofungin versus voriconazole included nine *Fusarium* cases and showed baseline imbalance, leaving organism-specific estimates exploratory (Atighehchian et al., 2025). The original Rose Bengal Electromagnetic Activation with Green Light for Infection Reduction trial found no overall six-month benefit from adjunctive rose-bengal photodynamic therapy; an exploratory estimate in *Fusarium* favored treatment, although the 95% confidence interval included no effect (Prajna et al., 2026b). At 12 months, the prespecified follow-up found no benefit in visual acuity, infiltrate or scar size, or corneal perforation/therapeutic keratoplasty, and no subgroup signal by organism (Prajna et al., 2026a). The pilot comparison and rose-bengal trial do not support routine organism-specific use. Advancement of host-directed or combination approaches requires direct antifungal activity, stromal exposure, tissue preservation, and clinically meaningful benefit in the relevant pathogen and disease stage.

### 6.3 Deep invasion and treatment failure

Treatment failure may reflect persistent spatial infection, tissue injury, inadequate effective exposure, or several of these processes together; a high MIC is only one possible contributor. Direct spatial evidence remains sparse and derives mainly from surgical specimens. **Table 2** includes a late surgical specimen showing diffuse full-thickness invasion by *F. falciforme*. In a 174-case lamellar keratoplasty series, vertical hyphal growth was strongly associated with postoperative recurrence compared with lamella-parallel growth; historical species labels and surgical selection confine this result to a spatial recurrence signal, not an FSSC-member effect (Xie et al., 2008). *Fusarium*-positive therapeutic keratoplasty transcriptomics captured shared and pathogen-associated host-response programs in a small cohort selected through treatment failure and surgery (Dinesh et al., 2025). A five-case surgical series likewise represented rapid progression after medical nonresponse, outside the untreated history of invasion (Oliver-Gutierrez et al., 2024). Measurements in late therapeutic tissue and anterior-chamber exudate differed by compartment: drug concentration did not consistently correspond to MIC or clinical response, and anterior-chamber culture, corneal-button culture, and histology yielded different results (Kaur et al., 2025; Patil et al., 2025). A single exposure measure or specimen type cannot account for treatment failure.

Clinical trials and treatment syntheses use endpoints that do not measure spatial fungal persistence directly. Recent antifungal-treatment syntheses evaluate visual acuity, healing or microbiological response, perforation, and therapeutic keratoplasty, without measuring hyphal depth or viable stromal burden (Hoffman et al., 2022). The *Fusarium* subgroup analysis of the second Mycotic Ulcer Treatment Trial likewise used perforation or therapeutic keratoplasty as its principal failure outcome and did not map intracorneal fungal distribution (Prajna et al., 2017). A sequence-resolved case attributed to *Fusarium tonkinense* combined marked antifungal resistance with destructive progression, but one case cannot establish a member-level severity hierarchy or show that resistance caused deep invasion (Schrecker et al., 2021). Therapeutic keratoplasty is simultaneously a clinical outcome and a tissue source: it records uncontrolled disease while selecting the late specimen in which invasion, exposure, and host injury are measured. A recent synthesis of transplantation outcomes in infectious keratitis shows that anatomical control, graft survival, recurrence, and visual recovery are distinct postoperative outcomes (Onofrei et al., 2026). Therapeutic keratoplasty marks failure of disease control; it does not measure isolate virulence itself.

Cohort and model data impose further qualifications. Canadian multicenter data showed different risk factors and outcomes for yeast and filamentous keratitis, so mixed-pathogen estimates cannot define FSSC-specific mechanisms of failure (Ling et al., 2024). A rabbit model enabled serial scoring, histology, and fungal-burden comparison after delayed luliconazole or natamycin treatment of infection caused by the strain reported as *Fusarium solani*, but did not reproduce human visual recovery (Arimoto et al., 2023). Perforation, globe retention, recurrence, and final vision are separate clinical outcomes. A comprehensive account of treatment failure requires authenticated identity, spatial invasion, viable burden, developmental state, tissue drug exposure, host-mediated damage, treatment timing, and patient outcome. Available prospective and retrospective cohorts have yet to measure this sequence in the same patients with FSSC keratitis. A more informative prospective design would pair multilocus-authenticated isolates with spatial pathology, viable burden, compartment-specific drug concentrations, host-response measurements, and prespecified longitudinal endpoints.

## 7 Discussion

### 7.1 Isolate identity and severe disease course

Ocular isolate collections vary markedly in taxonomic resolution (**Table 1**). Historical *F. solani* labels encompass lineages and isolates identified at different levels; current nomenclature improves traceability, but pathogenic behavior still requires direct testing (de Hoog et al., 2023; Unalan-Altintop and Arikan-Akdagli, 2025). **Table 2** shows that authenticated FSSC isolates differ in adhesion, epithelial invasion, tissue penetration, and host-cell damage in defined ocular systems. Other studies describe isolate- and maturation-dependent biofilm phenotypes accompanied by reduced antifungal susceptibility (Zhang et al., 2012, 2024). Across these studies, isolate-level variation is evident in several traits, although those traits have not been measured uniformly across all members. Taxonomy identifies the biological unit under study; functional experiments determine how it behaves. A stable member-level ranking of severe disease would require both.

Host biology and experimental design shape how isolate-level traits are expressed. Mechanistic studies show that receptor engagement, myeloid-cell state, fungal clearance, and tissue preservation can diverge, while epithelial integrity, fungal phase, inoculation route, tissue architecture, host species, and treatment timing alter model performance (**Table 3**). The same trait may become prominent, attenuated, or qualitatively different in another host or model. Observations from a monolayer, ex vivo cornea, or animal model cannot be generalized automatically to all FSSC members or translated directly into patient severity. The strongest inference comes from measuring isolate identity, viable burden, spatial tissue injury, and host response within the same experimental platform.

Clinical studies rarely connect these elements within the same patient. The contact-lens outbreak resolved substantial genotype diversity but did not link individual genotypes to clinical courses (Chang et al., 2006). A recent tertiary-referral cohort characterized microbiology, treatment, and outcomes without paired isolate-level functional phenotyping in corneal systems (Yu et al., 2025). Susceptibility–outcome studies associated resistant phenotypes with selected clinical outcomes but did not identify the invasive mechanism involved (Vishwakarma et al., 2025). Experimental models offer detailed functional readouts yet lack matched longitudinal patient data, host-response measurements, and compartment-specific drug exposure. Late surgical tissue adds spatial and immunological information, but treatment failure determines its selection and untreated progression cannot be reconstructed. Treatment escalation, perforation, and poor vision record loss of disease control; they do not identify the fungal mechanism responsible. Available evidence supports pathogenic heterogeneity among isolates without establishing a reproducible member-level severity ranking. Such a ranking would require prospective linkage of the same authenticated isolate to corneal behavior, host injury, treatment exposure, and patient outcome across diverse clinical populations.

### 7.2 Causes of inconsistent findings

Nomenclature and sampling can generate apparent discrepancies before biological effects are considered. Name changes alter reporting units even when the underlying isolates remain unchanged, making it important to retain links between historical and current designations (Borman et al., 2025). Global estimates vary with geography, access to care, microbiological capacity, and diagnostic ascertainment; cohorts assembled in these settings represent different study populations (Brown et al., 2021a). Mixed-source collections combine human clinical, animal, and environmental *Fusarium* or *Neocosmospora* isolates, and corneal inferences depend on explicit separation by provenance (Saenz et al., 2025). Standard laboratory strains, clinical corneal isolates, and mixed-source panels should likewise be treated as distinct biological groups unless provenance, passage history, and experimental reuse are documented.

Discordance also arises from differences in outcome and exposure definitions. Recent clinical syntheses and tertiary-referral data show that studies variously prioritize ulcer size, final visual acuity, perforation, surgery, microbiological cure, or treatment escalation; these outcomes do not directly measure isolate invasion (Ghenciu et al., 2024; Yu et al., 2025). Among trial isolates, natamycin and voriconazole MICs were associated with selected outcomes, but the relation varied across endpoints (Sun et al., 2014). One randomized study compared natamycin alone with natamycin plus topical voriconazole while both groups received oral ketoconazole, so its estimate is not equivalent to a comparison of topical monotherapies (Di Zazzo et al., 2025). Formulation, residence time, and corneal penetration can also change effective exposure without any change in the organism (Mascarenhas et al., 2023).

Model choice, disease stage, and host state add further sources of variation. Recent cornea-chip synthesis shows that fluid dynamics, barrier architecture, cell composition, and mechanical forces can alter the tissue phenotype being measured (Kim et al., 2025). Surface challenge after abrasion and intrastromal inoculation represent different stages of entry; cell assays, ex vivo tissue, animal scores, and post-treatment keratoplasty specimens likewise sample different compartments and phases of disease. Host factors also vary. Diabetes or immune compromise may alter presentation, while topical corticosteroid exposure or subsequent steroid management may change progression and inflammatory appearance (Knutsson et al., 2021; Bisen et al., 2024). Ocular antifungal immunity may reduce fungal burden while worsening tissue injury, which makes separate assessment of viable burden, inflammation, and transparency essential (Abbondante et al., 2023). Cross-study comparison requires joint reporting of taxonomy, source, inoculation route, fungal phase, host state, treatment history, exposure compartment, sampling time, and endpoint definition. Residual differences may arise from isolate biology, host biology, methodological variation, or their interaction.

### 7.3 Clinical translation and future research priorities

In current practice, isolate-level heterogeneity supports preserving and authenticating cultured isolates and interpreting molecular identity together with imaging-defined invasion depth rather than using a broad FSSC label alone (Huang et al., 2022b; ElZawahry et al., 2026). Deep or vertically oriented stromal invasion may indicate a higher-risk disease course, including recurrence after lamellar keratoplasty, although this spatial pattern does not by itself establish intrinsic isolate virulence (Xie et al., 2008). Antifungal susceptibility results may inform treatment selection but should be integrated with tissue penetration, prior therapy, and host-mediated injury; current evidence does not yet support routine isolate-specific prognostic scores or treatment algorithms (Huang et al., 2022b; Patil et al., 2025).

Future studies will be more informative when cohorts are comparable, even at the cost of smaller sample sizes. Multiregional consortia could assemble corneal-isolate collections using shared eligibility criteria, multilocus authentication, common reference databases, and standardized provenance metadata. Corneal isolates should remain separate from nonocular sources, with pretreatment status, specimen type, referral pathway, storage interval, passage history, and reuse recorded prospectively. For contact-lens users, including patients undergoing overnight orthokeratology for myopia control, future cohorts should also record lens type, overnight wear, hygiene, water exposure, and disinfection practices, because these variables may modify the exposure context but should not be conflated with the intrinsic virulence of the recovered isolate (Chang et al., 2006; Kam et al., 2017). *Fusarium* reference genomes demonstrate substantial core-genome variation (Gomez-Chavarria et al., 2024); keratitis studies can now pair genomic data from the same authenticated isolates with adhesion, epithelial entry, stromal invasion, biofilm formation, hyphal susceptibility, and tissue-damage phenotypes. Candidate traits such as thigmotropism warrant further study once they can be tested reproducibly in authenticated keratitis isolates and structured corneal tissue (Rim et al., 2026). Before data collection, protocols should define sampling times, the handling of repeated isolates, and the clinical endpoint assigned to each comparison.

Model development should carry the same isolate through complementary systems that add tissue architecture and host components. Recent cornea-chip synthesis identifies multicellular barriers, controlled flow, and application-specific validation as priorities, providing an architectural starting point without direct FSSC validation (Kim et al., 2025). Resident stromal cells, perfusion, recruited myeloid cells, recoverable viable burden, and depth-resolved imaging would address major omissions in current systems. CRISPR/Cas9 gene replacement has been established in a keratitis isolate reported as *F. solani* var. *petroliphilum* (Lightfoot and Fuller, 2019). Defined parental, edited, and complemented strains could then be carried unchanged through epithelial culture, structured human corneal tissue, ex vivo corneas, and immune-competent animals using harmonized endpoints.

Human studies also require spatial and pharmacological calibration. Dual-target nucleic-acid amplification for *F. keratoplasticum* supplies organism-directed molecular evidence, while combined IVCM and AS-OCT supports structural localization and longitudinal monitoring; neither approach alone yields a depth-resolved map of viable fungus (Chen et al., 2026b; ElZawahry et al., 2026). A cutinase-antibody assay detected *F. solani* in cell and animal experiments and remains a preclinical antigen-directed candidate without validated diagnostic accuracy in patients (Jo et al., 2025). Combining imaging with culture or molecular estimates of viability and authenticated-isolate data would connect tissue structure with organism status. Aqueous-humor voriconazole measurements establish exposure in that fluid compartment (Thiel et al., 2007). Prospective studies should add depth-resolved stromal concentrations and align them with spatial fungal burden, treatment timing, host-response markers, and prespecified visual, perforation, and surgical outcomes. Whenever clinically feasible, sampling should precede major treatment changes. Tissue obtained later at surgery should be treated as selected material from advanced disease, not as a baseline specimen.

Reproducibility also depends on deposited strains and linked data. Culture-collection resources such as GCM/gcType and FUSARIUM-ID show how living strains, sequences, taxonomy, growth conditions, provenance, and database versions can be connected through persistent accessions (Torres-Cruz et al., 2022; Fan et al., 2025). Sequence files, phenotype protocols, images, and clinical metadata should use persistent identifiers and interoperable records consistent with the principles of findability, accessibility, interoperability, and reusability (Wilkinson et al., 2016). Repositories should document strain passage, storage, and experimental reuse. This allows technical failure to be distinguished from biological discordance and prevents repeated use of one strain from being mistaken for independent replication.

### 7.4 Conclusion

Current evidence supports isolate-level pathogenic heterogeneity in FSSC keratitis but not a reproducible species- or member-level hierarchy of virulence or clinical severity. Taxonomic assignment, fungal developmental state, corneal compartment, host response, drug exposure, and model design should be measured separately and linked prospectively. The most informative next step is a matched isolate–model–patient design in which the same multilocus-authenticated clinical isolate is followed through spatial corneal phenotyping, viable-burden assessment, host-injury profiling, compartment-specific pharmacology, and longitudinal outcomes. This approach would distinguish persistent viable infection from inadequate tissue exposure and host-mediated damage, improve interpretation across biological models, and provide a stronger basis for prognostic markers and mechanism-informed treatment.
